# Electrocardiographic findings of methanol toxicity: a cross-sectional study of 356 cases in Iran

**DOI:** 10.1186/s12872-020-01691-y

**Published:** 2020-09-14

**Authors:** Mohammad Hossein Nikoo, Alireza Arjangzadeh, Maryam Pakfetrat, Shahrokh Sadeghi Boogar, Vahid Mohammadkarimi, Vahid Reza Ostovan, Zohre Khodamoradi, Jamshid Roozbeh, Mohammadreza Khalili, Farnaz Kamali Haghighi Shirazi, Paryia Kouhi, Seyed Taghi Heydari

**Affiliations:** 1 Non-communicable Disease Research Centre, Shiraz University of Medical Sciences, Shiraz, Iran; 2 Cardiology Department, Shiraz University of Medical Sciences, Shiraz, Iran; 3 Department of Internal Medicine, Shiraz Nephro-Urology Research Center, Shiraz University of Medical Sciences, Shiraz, Iran; 4 Department of Internal Medicine, School Of Medicine, Shiraz University of Medical Sciences, Shiraz, Iran; 5 Clinical Neurology Research Center, Shiraz University of Medical Sciences, Shiraz, Iran; 6 Poostchi Eye Research Centre, Ophthalmology Department, Shiraz University of Medical Sciences, Shiraz, Iran; 7 Health Policy Research Center, Institute of Health, Shiraz University of Medical Sciences, Building No.2.8th Floor School of Medicine Zand Avenue, P.O.Box:71345-1877, Shiraz, Iran

**Keywords:** Methanol toxicity, ECG, Myocardial infarction, Iran

## Abstract

**Background:**

Methanol is widely used in industry; however, methanol poisoning is not common. In this regard, a number of outbreaks have been recently reported due to inappropriate processing of alcoholic beverages. Shiraz, a city located in the southern part of Iran, faced one of such outbreaks in 2020 during COVID-19 pandemic. There is no sufficient literature on the electrocardiographic findings in methanol toxicity. This study aimed to address this gap in the literature.

**Method:**

A total of 356 cases with methanol toxicity referred to Shiraz University of Medical Science Tertiary Hospitals (Faghihi and Namazi) in March and April, 2020. The clinical findings of blindness and impaired level of consciousness, lab data such as arterial blood gas, electrolytes, and creatinine, and the most common findings from ECGs were collected.

**Results:**

The most common ECG findings were J point elevation (68.8%), presence of U wave (59.2%), QTc prolongation (53.2% in males and 28.6% in females), and fragmented QRS (33.7%). An outstanding finding in this study was the presence of myocardial infarction in 5.3% of the cases. This finding, to the best of our knowledge, has only been reported in a few case reports. Brugada pattern (8.1%) and Osborn wave (3.7%) were the other interesting findings.

In multivariate analysis, when confounding factors were adjusted, myocardial infarction, atrioventricular conduction disturbances, sinus tachycardia, and the prolonged QTC > 500 msecond were four independent factors correlated with methanol toxicity severity measured with arterial blood PH on arterial blood gas measurements, with odds ratios of 12.82, 4.46, 2.32 and 3.15 (*P* < 0.05 for all), respectively.

**Conclusion:**

Electrocardiographic variations during methanol intoxication are remarkable and well-correlated with poisoning severity. Myocardial infarction was an egregious and yet a common concerning finding in this sample, which need to be ruled out in methanol toxicity.

## Research background

Methanol is an odorless alcohol with industrial application, especially in solvents [1–3]. Most of the intoxication events incidentally occurs by children and rarely as a suicidal attempt. The real toxic material is formic acid, which is a metabolite of methanol in human body and its half time is around 30 h [3]. The slow rate of metabolism of formic acid is the main cause of delayed presentation of methanol toxicity [4]. Clinical findings are blindness, renal shutdown, brain damage, and finally death, if left untreated [2, 3]. Treatments often include dialysis and ethanol or fomepizole [2]. The outbreaks of methanol toxicity, defined as three cases presenting within 72 h, have been increasingly occurred in recent years [4]. These are possibly due to the inappropriate distill or fermentation of alcoholic beverages [5]. Common and novel misconceptions about the protective and therapeutic role of alcohol consumption for COVID-19 have unfortunately contributed to this public health problem in Iran. As a result, and given the forbidden status of alcohol in Iran, the availability of homemade alcohol, which is sometimes contaminated with methanol, has increased, leading to enhanced rates of methanol toxicity [6, 7]. After the first half of March 2020, Shiraz University of Medical Sciences (SUMS) faced the methanol toxicity outbreak, which was roughly associated with the first days of Covid-19 pandemic; however, it was nearly faded in second decade of April. The reported numbers as well as mortality and morbidity rates were shockingly high.

Cardiology service was involved in the care of these patients and provided valuable data about their management and their ECG findings. To date, Jaff’s et al. study on nine patients with methanol toxicity in 2014, and Sanaei-Zade’s et al. (2013) study on 42 were two studies with the largest sample sizes analyzing the ECG findings attributed to methanol toxicity [8, 9]. Accordingly, this study is the largest study reporting the ECG findings from methanol toxicity. This study aimed to describe the most common ECG findings among patients with methanol toxicity and their association with the severity of intoxication.

## Methods

This is a cross-sectional study on 356 patients with methanol intoxication and were referred to Shiraz University of Medical Science Tertiary Hospitals (Namazi & Faghihi) in March and April, 2020. The data were collected from the available charts by a cardiologist. The data included demographic characteristics, history, physical examination findings, lab data, and ECG (by Cardiax PC ECG). Then the ECGs were reviewed and reported by two cardiologists. The following patients met the inclusion criteria in this study: All patients who used the known alcoholic beverage with proved methanol impurity and developed expected clinical findings. Exclusion criteria were previous myocardial infarction, supra-ventricular or ventricular arrhythmias, CABG, any known genetic cardiac disease such as Brugada, long QT syndrome, and short QT syndrome.

The history and clinical data included the chronicity of alcohol usage, use of other substances, cardiac and non-cardiac comorbidities, blindness, altered level of consciousness, and death and GCSS (Glasgow Coma Score Scale) for altered level of consciousness. Gathered Laboratory variables were arterial blood gas measurements, renal function measures, electrolytes, and blood sugar.

There are some reports on the following ECG parameters: some basic interpretations (rhythm, rate, axis, hypertrophy and enlargement), relatively new findings on methanol toxicity (ST elevation myocardial infarction and atrioventricular conductance disturbances), repolarization variants (J elevation, early repolarization [10], Brugada pattern [11], U wave, QTc prolongation, QT dispersion (QTD), the slope of terminal part of T wave (TTerm SL) [12], and Osborn wave [13]), and depolarization abnormalities (Bundle Branch Block, low voltage QRS, poor R wave progression, and fragmented QRS [14]).

Myocardial infarction was defined as typical ST elevation in a group of ECG leads, which face the corresponding myocardial wall with evolutionary changes and is associated with a raise in troponin level and chest pain complaint [15]. Moreover, the definition for renal failure is based on creatine above 1.4 mg/dl [16].

The patients in the present study were categorized according to Hassanian-Moghaddam et al., who introduced PH < 7 as a marker of poor prognosis and severe acidosis [17]. The focus of this study is on the ECG findings of a large population of the patients with methanol toxicity. In this study, PH < 7 (measured by OPTI medical blood gas and electrolyte analyzers) was called severe acidosis and used as an index of severity for methanol toxicity. The association between acidemia and ECG findings were studied using univariate and multiple variables for a logistic regression model.

### Statistical analysis

Statistical Package for the Social Sciences Version 21.0 (SPSS Inc., Chicago, IL, USA) was used to analyze the data. Frequency (%) was used for categorical variable such as sex, alcohol dependency, comorbidity, ECG finding. Also, mean ± standard deviation was used for age and laboratory finding. Chi-square test was used to assess the relationships between PH and ECG variables in methanol toxicity. Furthermore, odds ratio (OR) and corresponding confidence interval (95% CI) was calculated by univariate logistic regression. Multiple logistic regression was performed to determine the independent relationship between PH and ECG variables. *P* < 0.05 was considered to be statistically significant.

## Results

Among the 356 patients, 328 (89.9%) persons were male, and male/female ratio was 9.2. The mean age of the participants was 32.76 ± 10.61 with an age range of 15–72 years old. Moreover, 44.7% of the participants used alcohol regularly, at least once a week; hence, more than half of the patients used alcohol infrequently or for the first time. The concurrent use of the others substances was observed in 9.5% of the participants, with opium as the most common concurrently used substance. Comorbidities were also observed in 12% of the patients (6.5% cardiac and 5.6% non-cardiac cases). The mortality rate in our academic and well-experienced centers to manage arrhythmia and other cardiac complications reached 16.6% (59 persons). The most common clinical presentation was visual impairment, as reported for 251 persons (70.5%). The sinus rhythm was observed in 95.8% of the participants, and only 4.2% of these individuals were found to have other rhythms such as atrial fibrillation and low atrial rhythm (Table 1). Arterial blood gas and biochemical profile were also measured, and the reports were summarized in Table 2.

**Table 1:** Clinical variables and ECG findings among methanol toxicity

			Frequency	Percent
**Alcohol dependency**			142	39.9
**Other substance abuse**			32	9.0
**Comorbidity**	Cardiac	HTN	18	5.1
		PCI	4	1.1
		PTE	1	0.3
	Non-cardiac	DM	6	1.7
		Seizure	2	0.6
		Probably Asthma	3	0.8
		Malignancy	2	0.6
		Psychiatry	2	0.6
		Hemophilia	1	0.3
		Down syndrome	1	0.3
		Fatty liver	1	0.3
		Splenectomy	1	0.3
		Hypothyroid	1	0.3
**Decreased visual acuity**			251	70.5
**Death**			59	16.6
**Renal failure**			137	38.5
**GCSS**	GCSS 15		252	72.6
	GCSS 3		48	13.8
	GCSS 4–14		47	13.5
**Rhythm**	Sinus		341	98.0
	Atrial fibrillation		3	0.9
	Low atrial rhythm		2	0.6
	Accelerated idio-ventricular rhythm		2	0.6
**Rate**	Normal		251	71.3
	> 100		89	25.3
	< 60		12	3.4
**Axis**	Normal		296	83.5
	Right		36	10.1
	Left		19	5.3
	Extreme		4	1.1
**Hypertrophy and enlargement**	RV		9	2.6
	RA		8	2.3
	LV		6	1.7
	LA		9	2.6
	Mixed		8	2.3
**Infarction**			19	5.5
**AVB**	First degree		11	3.2
	Third degree		1	0.3
**J point elevation**			245	70.6
**Brugada pattern**	Type 1		4	1.2
	Type 2		3	0.9
	Type 3		13	3.7
**Early repolarization**			168	48.4
**Osborn wave**			13	3.7
**QTc**	<=500		278	80.1
	> 500		69	19.9
**QTd**	< 40		179	51.6
	> = 40		168	48.4
**Fragmented QRS**			120	34.7
**BBB**	RBBB		24	7.0
	LBBB		2	0.6
	IVCD		2	0.6
**U wave**			212	61.3
**Poor R progression**			14	7.0
**Low voltage**			15	4.3

*HTN* Hypertension, *DM* Diabetes Mellites, *PCI* Percutaneous Coronary Intervention, *PTE* History of Pulmonary Thrombo-Emboli, *BBB* Bundle branch block, *STEMI* ST segment elevation myocardial infarction, *AVB* Atrioventricular conduction block

**Table 2:** Laboratory finding in methanol toxicity

Variables	N	Mean	Standard Deviation
**PH**	349	7.14	0.22
**Bicarbonate (meq/l)**	349	11.14	8.02
**Partial Pressure of Carbon Dioxide (torr)**	349	27.82	13.81
**Oxygen saturation (%)**	337	90.50	10.43
**Blood urea nitrogen (mg/dl)**	342	14.18	10.83
**Creatinine (mg/dl)**	342	1.43	0.63
**Sodium (meq/l)**	337	142.17	4.21
**Potassium (meq/l)**	332	4.83	1.17
**Calcium (mg/dl)**	251	9.55	0.77
**Magnesium (mg/dl)**	177	2.41	1.55
**Blood sugar (mg/dl)**	311	143.97	95.85

### Univariate analysis

In the univariate analysis, the patients with QTc > 500 had more serious acidosis (OR = 3.92; 95% CI: 2.17–7.07; *P* < 0.001). This higher acidotic PH was observed in QTD > 40 (OR = 1.80;95% CI: 1.05–3.09; *P* = 0.032), Atrioventricular block (OR = 9.48; 95% CI: 2.76–32.55, *P* < 0.001), sinus tachycardia (OR = 2.03; 95% CI: 1.14–3.63, *P* < 0.026), Brugada pattern (OR = 3.30; 95% CI: 1.27–8.57; *P* = 0.01), ST elevation Myocardial Infarction (OR = 9.93; 95% CI: 3.57–27.58, *P* < 0.001), and Bundle Branch Block (OR = 2.86,95% CI: 1.24–6.63; *P* = 0.011). Furthermore, there was no relationship between severe acidosis with T slope, J elevation, poor R wave progression, and low voltage QRS (*P* > 0.05) (Table 3).

**Table 3:** Association between PH and ECG variables in methanol toxicity with univariate and multiple logistic regression

		PH		***P***-value	Odds ratio (95% CI) Univariate	***P***-value	Odds ratio (95% CI) Multiple	***P***-value
		> 7	<=7					
**QTC**	< 500	238 (85.6)	40 (14.4)	< 0.001	1	–	1	–
	> = 500	41 (60.3)	27 (39.7)		3.92 (2.17–7.07)	< 0.001	3.15 (1.55–6.40)	0.001
**QT dispersion**	< 40	153 (85.0)	27 (15.0)	0.032	1	–	1	–
	> = 40	126 (75.9)	40 (24.1)		1.80 (1.05–3.09)	0.034	1.19 (0.62–2.26)	0.603
**T slope**	< 69	184 (81.1)	43 (18.9)	0.700	1	–	1	–
	> = 70	96 (79.3)	25 (20.70		1.11 (0.64–1.93)	0.700	2.28 (1.11–4.69)	0.488
**AVB**	No	275 (82.6)	58 (17.4)	< 0.001	1	–	1	–
	Yes	4 (33.3)	8 (66.7)		9.48 (2.76–32.55)	< 0.001	4.46 (1.03–19.23)	0.045
**Rate**	60–100	208 (84.2)	39 (15.8)	0.026	1	–	1	–
	< 59	8 (66.7)	4 (33.3)		2.67 (0.77–8.29)	0.123	1.8 (0.371–8.70)	0.467
	> 100	63 (72.4)	24 (27.6)		2.03 (1.14–3.63)	0.017	2.32 (1.19–4.53)	0.014
**Brugada**	No	268 (82.0)	59 (18.0)	0.010	1	–	1	–
	Yes	11 (57.9)	8 (42.1)		3.30 (1.27–8.57)	0.014	2.91 (0.94–8.99)	0.063
**J elevation**	No	87 (85.3)	15 (14.7)	0.156	1	–	1	–
	Yes	192 (78.7)	52 (21.3)		1.57 (0.84–2.94)	0.159	1.16 (0.55–2.48)	0.693
**STEMI**^a^	No	273 (83.2)	55 (16.8)	< 0.001	1	–	1	–
	Yes	6 (33.3)	12 (66.7)		9.93 (3.57–27.58)	< 0.001	12.82 (3.82–43.11)	< 0.001
**BBB**^a^	No	261 82.1)	57 (17.9)	0.011	1	–	1	–
	Yes	16 (61.5)	10 (38.5)		2.86 (1.24–6.63)	0.014	2.48 (0.92–6.66)	0.073
**Poor progression**	No	260 (81.5)	59 (18.5)	0.101	1	–	1	–
	Yes	17 (68.0)	8 (32.0)		2.07 (0.86–2.03)	0.107	2.41 (0.88–6.56)	0.086
**Low voltage**	No	267 (80.9)	63 (19.1)	0.398	1	–	1	–
	Yes	10 (71.4)	4 (28.4)		1.70 (0.52–5.58)	0.385	1.93 (0.50–7.54)	0.343

^a^*BBB* Bundle branch block, *STEMI* ST segment elevation myocardial infarction, *AVB* Atrioventricular conduction block, *P* value less than 0.05 considered significant

### Multivariate analysis

In multiple logistic regression, there was a statistically significant relationship between severe acidosis (PH < 7) with QTc > 500 (OR = 3.15, CI = 95%: 1.55–6.40; *P* = 0.001), Atrioventricular block (OR = 4.46; CI 95%: 1.03–19.23; *P* = 0.045), sinus tachycardia (OR = 2.32; CI 95%: 1.19–4.53; *P* = 0.014), and ST elevation myocardial infarction (OR = 12.82; CI 95%: 3.82–43.11; *P* < 0.001) (Table 3).

## Discussion

**Fig. 1:**
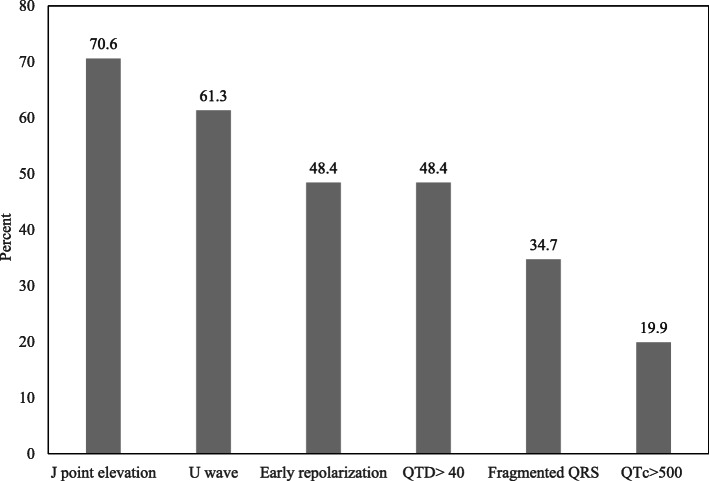
The most common ECG findings in methanol toxicity

Methanol intoxication, when sever enough, causes many electrocardiographic changes. J point elevation and U wave are most frequent (Fig. 1), While others like QTc more than 500 m-second tends to be an index of severity (Table 3).

A total number of 356 patients were included in this study, a majority of whom were male. This can be explained by the participants’ cultural background and relevant social stigma of alcohol usage for women in Iran. Compared to previous outbreaks [18, 19], the age distribution of the population was broader as the study included teenagers as well as senior patients who were in their 70’s in some cases. The involvement of the youths may be a matter of concern for social activists in Iran.

In this study, interesting finding were myocardial infarction, AVB and sinus tachycardia, and QT > 500, all of which predispose the patients to arrythmia. The mechanism of arrythmia is tightly related to acidosis. Acidosis is an obvious biochemical marker with regard to its several mechanisms. Formic acid makes a pivotal contribution to the PH drop, which causes hyperkalemia when acidosis makes potassium to shift out of cells. Another reason for high potassium level may be oliguria, which prevent potassium loss in urine. Another consequence of acidosis seems to be hypercalcemia caused by a decrease in binding to protein under this condition. The extra amount of both ions contributes to arrhythmia mechanism [20].

Different mortality rates have been reported for methanol intoxication. The reason may be controversy in definitions. Taiwan nationwide survey showed 40% long-term mortality [21]; however, a study from UK reported a 11% rate [8]. Another research with the rate of 40% was performed by Sanaei-Zade et al. [9]. The mortality rate in our series was slightly < 17%, which seems to be more comparable to a study by Jaff et al. [8]; however, this is an in-hospital mortality in two referral centers, and the ones related to the outpatient cases were not included. The mortality and ocular and brain damage rates are in agreement with some previous outbreak reports [19, 22–24].

Although the most common finding in a previous report from the UK [8] was sinus tachycardia, most cases in this study seemed to have normal heart rate; however, the tachycardia proved to be a severity index in the present research. In this regard, the lower prevalence may be due to less severe intoxication in our patients. The heart rate > 100 was observed in 25.3% of the sample, which was nearly six times as great as bradycardia (rate < 60). In other words, about three quarter of our cases were at a normal range in this regard.

Although there are few case reports on the myocardial infarction in methanol toxicity [23], our survey was the first study, which could determine the prevalence of this complication. However, the exact pathophysiologic mechanism yet requires further research. We proposed the following hypotheses: (1) This infarction is possible in relatively young patients with no usual risk factors for atherosclerosis [24]; (2) severe acidosis causes bleeding due to a decrease in fibrinogen level. In-situ thrombosis may sometimes occur with ongoing disseminated intravascular coagulation and possible myocardial infarction [25]; (3) Possible pathophysiologic mechanism may be endothelial dysfunction, which is a known entity in reduced extracellular PH, however such a dysfunction will end in vasodilation frustratingly [26]; and (4) Acidosis causes vasodilatation; thus, spasm, as a mechanism of myocardial infarction cannot be blamed [27]. Such hypotheses necessitate further investigation and research.

In our study, J point elevation was the most prevalent finding; therefore, repolarization abnormalities were to be categorized as early repolarization, Brugada phenocopy, and QT prolongation syndrome. According to the literature, arrhythmia can be predicted in accordance with the ECG findings such as QT prolongation, Brugada pattern, negative terminal portion of P wave in V1, inter-atrial block, and so on [28]. Another newly introduced observation on ECG is QRS fragmentation, which is correlated with mortality in myocardial infarction in recent surveys [29]. The only variable which was consistently correlated with the severity of disease was QTc prolongation> 500. The QT prolongation was reported in mostly all the previous studies [e.g., [3, 30, 31]]. However, its significance could not be emphasized due to the lower volume in older reports. QTc prolongation is the most commonly-used indicator; however, prolongation< 500 msec. May be interpreted doubtfully. Accordingly, we approached the values > 500 as an independent marker of severity [32, 33].

We noted two type 1 Brugada ECG pattern in two brain-dead patients as terminal event before their arrest. This finding has been reported in some critical situations with deadly consequences. As an example, similar pattern has observed in some head injured patients before their death in Neurosurgery Intensive care unit. There are also reports of same pattern during Propofol infusion [34, 35].

The best independent ECG indicators of methanol toxicity severity were QTc > 500 and heart rate > 100. Interestingly, severe poisoning was strongly associated with myocardial infarction and atrioventricular block in our survey (Table 3).

### Research limitations

The research design is cross-sectional so that it suffers from lack of long-term perspectives. Our diagnosis on myocardial infarction was based on the ECG finding, evolutionary changes, and enzyme rising in those with chest pain or chest pain equivalents; however, it disregarded coronary angiography and cardiac MRI.

## Conclusion

This cross-sectional survey was conducted on 356 patients admitted to hospitals due to methanol toxicity and examined the most common ECG findings and their association with PH as a marker of methanol intoxication severity. In this study, myocardial infarction, AVB and sinus tachycardia, and QT > 500 were four independent markers of severity.

## Data Availability

The datasets used and/or analyzed during the current study available from the corresponding author on reasonable request.

## Abbreviations

COVID-19: Coronavirus
ECG: Electrocardiogram
SUMS: Shiraz University of Medical Sciences
BBB: Bundle branch block
STEMI: ST segment elevation myocardial infarction
AVB: Atrioventricular conduction block
msecond: Millisecond
UK: United Kingdom
TTerm SL: Slop of terminal part of T wave
GCSS: Glasgow coma score scale
QTc: Corrected QT
QTD: QT dispersion
HTN: Hypertension
DM: Diabetes Mellites
PCI: Percutaneous coronary intervention
PE: History of pulmonary Thrombo-emboli
CABG: Coronary artery bypass grafting
